# African health innovation systems: preface

**DOI:** 10.1186/1472-698X-10-S1-I1

**Published:** 2010-12-13

**Authors:** Calestous Juma

**Affiliations:** 1 Belfer Center for Science and International Affairs, John F. Kennedy School of Government, Harvard University, 79 John F. Kennedy Street, Cambridge, MA 02138, USA

## Preface

Concern over access to essential medicines has dominated international health policy debates over the last two decades. Much of this debate has focused on the role of intellectual property rights in either restricting or enabling developing countries to address persistent and emerging medical challenges. Much of this debate has focused on African countries which have borne higher disease burdens due in part to their low income levels.

These arguments and the associated policy prescriptions are guided by the view that Africa will remain a marginal player in the world of health innovation and will continue to rely on imported solutions. This collection of original papers provides a different prognosis.  They reveal an emergent “health innovation system” in Africa that is driven by a combination of local research, entrepreneurship and institutional adaptations.

The papers draw from experiences in Ghana, Kenya, Madagascar, Nigeria, Rwanda, South Africa, Tanzania, and Uganda to provide a clear view of the elements of the emerging African health innovation system.  First, they demonstrate that the emerging innovation systems are driven by local health concerns and not by external interests. Second, the case studies underscore the importance of investment in research and development (R&D) and the generation of solution-oriented knowledge. Third, the essays demonstrate the importance of creating incentives and building institutions that can help to facilitate the commercialization of research results. Finally, the case studies show that Africa’s health innovation systems are integrated into the global knowledge ecology and benefit from extensive international partnerships.

These essays have far-reaching implications for national and international health innovation policy. In addition to presenting research findings, the papers provide clear policy prescriptions on how African countries can help to strengthen their emerging innovation systems so that they can improve health outcomes while contributing to overall economic diversification. This volume is relevant to the health profession as well to those responsible for economic transformation.  It challenges African countries to come up with policies and institutions that facilitate medical innovation. If they do this for health innovation, they will learn critical policy lessons which they can apply in other sectors.

These essays will change the way international health cooperation is conducted. The traditional belief that it is not cost-effective or feasible to undertake basic health research in Africa will need to reconsidered. In fact, the case for R&D partnerships with African institutions will become a critical element in the successful commercialization of drugs of relevance to Africa. More specifically, the papers show the significant role that African universities and research institutes are playing as centers of discovery and policy champions. Excluding them from R&D can only delay the deployment of critical technologies.

The timing of the publication of these papers is particularly critical. First, they have come at a time when firms in industrialized countries are rethinking their global strategies, especially in relation to the location of new research and production facilities. These papers show that some African countries could be viable partners as they seek to become part of the global knowledge ecology. Second, African economies are expanding, and more attention is shifting to knowledge-based industries. Health-related technologies are clear candidates for international partnerships. Third, African countries are integrating their economies to create large regional markets that can better foster innovation and foreign direct investment. All African regional economic integration organizations have identified medical research as one of their priorities. Finally, these regional organizations consider business incubation through facilities such as science parks as important mechanisms for fostering industrial clustering and raising economic productivity.

These papers offer important lessons that can help to guide Africa and its international partners to complement 20^th^ century policies on access to essential medicines and technology with 21^st^ century approaches that focus on building health innovation systems. Those who take this route will find these papers highly valuable and timely.

## Competing interests

The authors declare that they have no competing interests.

